# Acute Primary Angle Closure Caused by Plateau Iris After Ritodrine Use in a Primigravid Woman Successfully Treated With Clear-Lens Extraction: A Case Report

**DOI:** 10.7759/cureus.22036

**Published:** 2022-02-08

**Authors:** Sayaka Yoshida, Masaaki Yoshida, Noriko Himori, Shiho Kunimatsu-Sanuki, Toru Nakazawa

**Affiliations:** 1 Department of Ophthalmology, Tohoku University Graduate School of Medicine, Sendai, JPN; 2 Department of Aging Vision Healthcare, Tohoku University Graduate School of Biomedical Engineering, Sendai, JPN; 3 Department of Ophthalmology, Nishikasai Inouye Eye Hospital, Tokyo, JPN; 4 Collaborative Program for Ophthalmic Drug Discovery, Tohoku University Graduate School of Medicine, Sendai, JPN; 5 Department of Retinal Disease Control, Tohoku University Graduate School of Medicine, Sendai, JPN; 6 Department of Advanced Ophthalmic Medicine, Tohoku University Graduate School of Medicine, Sendai, JPN; 7 Department of Ophthalmic Imaging and Information Analytics, Tohoku University Graduate School of Medicine, Sendai, JPN

**Keywords:** clear-lens extraction, pregnant woman, ritodrine, plateau iris, acute primary angle closure

## Abstract

Acute primary angle closure (APAC) is rare in young adults (those under approximately 40 years old) and pregnant women. Here, we report the case of a 37-year-old primigravid woman with APAC caused by plateau iris after the use of ritodrine (a β2 stimulator) that was successfully resolved by clear-lens extraction five months after delivery. The patient presented with pain in her right eye after ritodrine infusion for threatened premature labor at 23 weeks of gestation. Her visual acuity was 20/40, and her intraocular pressure (IOP) was 31 mmHg in her right eye. The patient was diagnosed as having APAC with plateau iris based on ultrasound biomicroscopy (UBM) findings of irido-angle touch, anterior dislocation of the ciliary process, and an absent ciliary sulcus. The effectiveness of treatment with pilocarpine eyedrops was limited, and argon laser peripheral iridoplasty did not succeed in reducing IOP. An immediate resolution was achieved with clear-lens extraction. IOP has since stayed within 14-16 mmHg without any medication for seven years. This is the first reported case of APAC complicated with plateau iris after ritodrine use in a pregnant woman. This condition is rare in young adults, making it difficult to diagnose; however, UBM can be of great help. In this case, clear-lens extraction led to a successful outcome. Our case suggests that attention should be paid to drug associations when APAC occurs with plateau iris.

## Introduction

Acute primary angle closure (APAC) is an ocular emergency that can cause blindness. APAC typically occurs in older females with shallow anterior chambers. Patients are often hypermetropic with enlargement of the crystalline lens. APAC is rare in adults under 40 years of age but is most prevalent in complications with plateau iris [1,2]. APAC in elderly patients is associated with mydriasis-inducing drugs, such as anticholinergic agents; however, the association of drugs and APAC in plateau iris remains unclear because the presence of a plateau iris can be overlooked when the patient is examined with only a slit lamp. To our knowledge, only five cases of drug-associated APAC with plateau iris in patients between the ages of 36 and 60 years have been previously reported. APAC during pregnancy is also rare. In this report, we describe a case of APAC after the use of ritodrine (a β2 stimulator) in a 37-year-old pregnant woman and its successful treatment with clear-lens extraction.

## Case presentation

**Figure 1:**
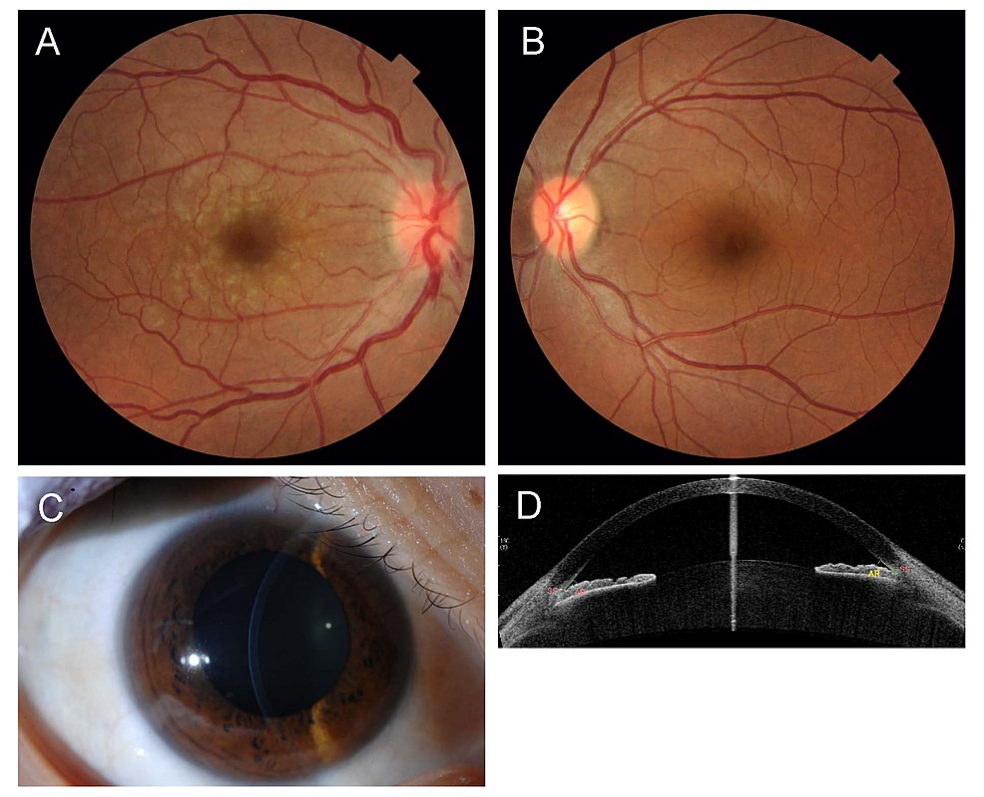
Photographs and imaging data at the first visit. A and B: Fundus images of the right and left eye, respectively, at the first visit to us. The right eye shows optic disc swelling with disc hemorrhage and retinal vein dilation and tortuosity, while the left eye shows a normal fundus. C: Slit-lamp photograph of the right eye showing a deep anterior chamber in the center. D: Anterior segment optical coherence tomography image of the right eye showing a deep anterior chamber in the center and shallow anterior chamber in the periphery.

A 37-year-old primigravid woman at 23 weeks of gestation presented with threatened premature labor and was admitted to the obstetrics and gynecology department of her local hospital. She received an intravenous infusion of ritodrine hydrochloride (1.5 mg/hour) to reduce uterine contractions and prevent premature labor. The next day, she experienced decreased visual acuity (VA) and severe pain in her right eye, and on the second day, she was seen by an ophthalmologist at her local hospital. Her VA was 20/40 (right) and 20/10 (left), and her intraocular pressure (IOP) was 31 mmHg (right) and 11 mmHg (left). She received treatment with 0.5% timolol eyedrops, 0.1% betamethasone eyedrops four times/day, oral acetazolamide 750 mg/day, and oral prednisolone 20 mg/day suspected with uveitic glaucoma. On the fourth day after admission, she was referred to us. IOP was normal at 7 mmHg (right) and 10 mmHg (left). However, the pupil of her right eye was moderately dilated and non-reactive. A slit-lamp examination revealed conjunctival hyperemia and diffuse corneal edema in the right eye. Fundus examination showed optic disc swelling with disc hemorrhage and retinal vein dilation and tortuosity in the right eye (Figure 1). Slit-lamp photography and anterior segment optical coherence tomography revealed that in both eyes, the anterior chamber was deep in the center and shallow toward the periphery (Figure 1).

**Figure 2:**
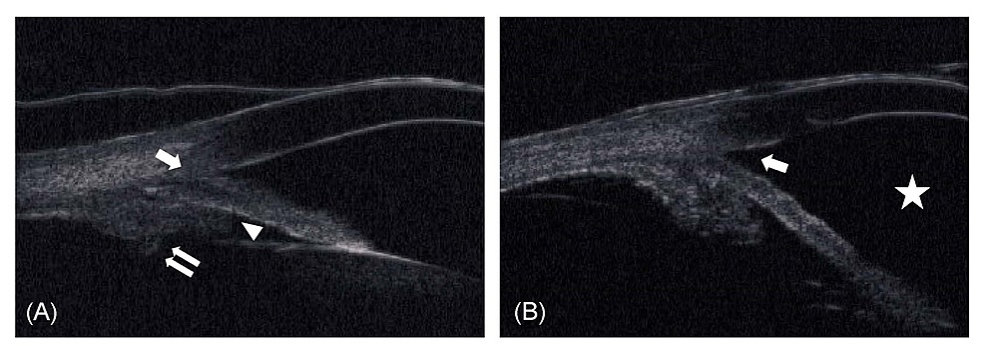
UBM images. A: Preoperative UBM image showing irido-angle touching (arrow), anterior dislocation of the ciliary process (double arrows), and an absent ciliary sulcus (arrowhead). B: Postoperative UBM image showing that the angle closure was resolved (arrow) and the anterior chamber had deepened (star). UBM = ultrasound biomicroscopy

Refraction was -0.25 diopters (D)/-0.75 D × 84° in the right eye; the left eye was emmetropic. Axial length was 21.88 mm in the right eye and 21.76 mm in the left eye. Anterior chamber depth was 2.32 mm in both eyes. Gonioscopy revealed a closed angle (Shaffer grade I) in both eyes. Peripheral anterior synechia (PAS) was not evident. Ultrasound biomicroscopy (UBM) revealed irido-angle touching, anterior dislocation of the ciliary process, and an absent ciliary sulcus (Figure 2).

Therefore, the patient was given a diagnosis of APAC with plateau iris. A course of pilocarpine hydrochloride treatment was begun in the right eye. On the 11th day after admission, the use of ritodrine was stopped because the threatened premature labor was well controlled.

**Figure 3:**
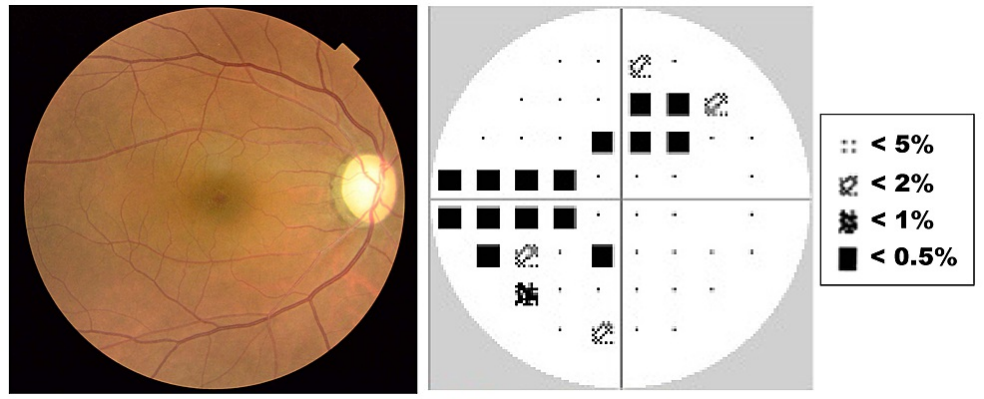
Fundus image and visual field seven years after APAC. A: Fundus image of the right eye seven years after APAC. The optic nerve head is slightly pale with cupping. B: The visual field as measured with the Humphrey field analyzer 24-2 seven years after APAC. Glaucomatous visual field defects are evident in the upper and inferior hemifield with -6.93 dB of mean deviation. APAC = acute primary angle closure

The treatment with pilocarpine eyedrops was effective for two weeks. Anti-glaucoma treatment with timolol eyedrops was stopped due to the possible negative effects on the unborn child. However, IOP increased again to 26 mmHg. Argon laser peripheral iridoplasty (ALPI) did not succeed in reducing IOP. Although we offered to perform clear-lens extraction as a treatment, the patient chose a course of IOP-control treatment with pilocarpine and anti-glaucoma eyedrops with 1% brinzolamide. Three months after the initial presentation, delivery was accomplished with a cesarean section. Two months later, the patient visited us with high IOP (39 mmHg) in the right eye, despite treatment with pilocarpine and brinzolamide eyedrops. The right eye showed PAS expanding to three quadrants. The pupil was non-reactive. We performed clear-lens extraction with one-piece intraocular lens insertion and goniosynechialysis (GSL) in the right eye. Two months later, we performed clear-lens extraction in the fellow eye to reduce the risk of APAC as much as possible after considering the fact that ALPI had not been effective in the right eye. We did not perform GSL in the fellow eye because gonioscopy revealed only 10-20% PAS and IOP had never increased in that eye. After the procedure, the angle closure was resolved and the anterior chamber deepened in both eyes (Figure 2). The patient has since reported no further episodes of pain, and bilateral IOP has stayed within 14-16 mmHg without any medication for seven years despite the residual PAS in her right eye. Her final VA was 20/20 (right) and 20/15 (left). Dilated fundus examination showed a slightly pale optic nerve head with cupping in the right eye, and the Humphrey field analyzer revealed a mild defect (Figure 3).

## Discussion

The etiology of APAC in young individuals differs from the older population and is typically associated with structural/developmental ocular anomalies such as plateau iris syndrome, rather than relative pupillary block. This makes the diagnosis of APAC difficult in this age group. Ritch et al. reported that the prevalence of plateau iris in young patients with angle closure (less than 40 years old) was 52.2% (35 of 67 patients) [1]. APAC occurs in high-risk individuals such as those with shallow anterior chambers or plateau iris triggered by mydriasis. Several drug classes, such as those with sympathomimetic activity or anticholinergic activity, have been reported to provoke the onset of APAC in patients with shallow anterior chambers; moreover, idiosyncratic reactions have also been reported with other drug classes [3,4]. However, reports of APAC with plateau iris due to pharmacological factors are rare. This patient developed APAC the day after administration of the β2 agonist ritodrine for threatened premature labor. The occurrence of APAC after the use of ritodrine has been previously reported in one case with a shallow anterior chamber in a pregnant woman [5]. Ritodrine does not induce mydriasis, leading the authors to consider that bed rest in a dark hospital room and the emotional stress of threatened premature labor induced mydriasis, resulting in APAC. However, the current case of APAC after ritodrine use raises the possibility that ritodrine itself may be a risk factor for APAC. We propose the hypothesis that the ciliary-body relaxing effect of β2 stimulation [6] might induce a change in the position of the ciliary body, leading to APAC in patients with a shallow anterior chamber or plateau iris. This hypothesis is supported by reported cases of APAC that developed after the inhalation of β2 stimulants (i.e., salbutamol or albuterol) in asthma patients, although an anticholinergic agent (ipratropium bromide) that induces mydriasis was also used with β2 agonists in these cases [7,8].

To our knowledge, only five cases of drug-associated APAC with plateau iris have been reported: a 40-year-old male receiving paroxetine for depression [9]; a 54-year-old female treated with paroxetine [10]; a 36-year-old female treated with topiramate [11]; a 60-year-old male treated with acetazolamide [12]; and a 45-year-old female treated with aripiprazole [13]. Table 1 summarizes these past cases and the current case. One eye of one of these previous cases underwent trabeculectomy and had a good final visual outcome [11], while seven eyes of four cases underwent laser peripheral iridotomy [9,11-13]. Thus, the case reported here is the first to have undergone clear-lens extraction for drug-associated APAC with plateau iris.

**Table 1:** Drug-associated APAC with plateau iris. APAC = acute primary angle closure; SSRI = selective serotonin reuptake inhibitor; UBM = ultrasound biomicroscopy; IOP = intraocular pressure; NA = not available; PI = peripheral iridotomy; LPI = laser peripheral iridotomy; ALPI = argon laser peripheral iridoplasty; TLE = trabeculectomy

Case	Age/Sex	Original disease	Drug	Laterality	Diagnosis	IOP R/L (mmHg)		Laser	Surgery
						Highest	Final		
Browning et al. [6] (2000)	40/M	Depression	Paroxetine (SSRI)	R	Gonioscopy, UBM	57/16	11/NA	B)YAG PI	-
Levy et al. [7] (2004)	54/F	Depression	Paroxetine (SSRI)	B	UBM	57/52	12/10	B)YAG PI	-
Rajjoub et al. [8] (2014)	36/F	Migraines	Topiramate (antiepileptic)	B	Gonioscopy	31/53	10-12/9-10	B)LPI	L)TLE
Man et al. [9] (2016)	60/M	Clinical study	Acetazolamide	B	UBM	36/35	12/11	-	-
Shen et al. [10] (2018)	45/F	Depression	Aripiprazole (atypical antipsychotic)	L	Gonioscopy	16/44	13/14	L)LPI, iridoplasty	-
Current case	37/F	Threatened premature labor	Ritodrine	R	UBM	46/14	14-16/14-16	R)ALPI	B)clear-lens extraction

Plateau iris is defined as an occludable angle in a gonioscopic examination, with a flat iris plane and a relatively deep central anterior chamber. UBM is of great help in diagnosing plateau iris because it can reveal the presence of other characteristics of the disease, such as an anteriorly positioned ciliary process, a narrow ciliary sulcus, a steeply rising peripheral iris, a downward angulation from the corneoscleral wall, and the presence of a flat iris plane. Eyes with plateau iris configurations are defined as those having at least two quadrants fulfilling these criteria [14,15]. In both eyes of our case, UBM revealed plateau iris in more than two quadrants. Recently, the presence of plateau iris has been reported to be a possible factor affecting disease severity and progression in severe primary angle closure glaucoma [16]. Thus, appropriate treatment of plateau iris is important. Moreover, plateau iris is likely underdiagnosed in the general population. In this case, the patient was suspected of having uveitis by the first doctor. In any patient, of whatever age, plateau iris should be carefully ruled out. The role of clear-lens extraction in adult-onset angle closure is well supported [17]. It follows that this procedure can also be applied in angle closure with plateau iris [18,19]. Rao reported resolution of bilateral APAC in a young 30-year-old female patient with plateau iris following clear-lens extraction [20]. This case also achieved a successful outcome with clear-lens extraction, even after medication and ALPI were not effective. Although clear-lens extraction for angle closure in young patients with plateau iris needs further investigation due to the risk of surgical complications, it can possibly resolve residual angle closure and reduce IOP.

Notably, this patient was a 37-year-old primigravid woman. The risk of APAC increases with age, and the average maternal age at first birth is increasing (in Japan, it increased from 27.0 years in 1991 to 30.7 years in 2019). Thus, APAC in pregnant women may become more common, especially after ritodrine use. It is important to consider the possibility of APAC due to plateau iris in cases of rising IOP in pregnant women as well as in all younger adults.

## Conclusions

This is the first case report of APAC with plateau iris after ritodrine use in a pregnant woman. This case showed that UBM can be of great help in diagnosing APAC in younger adults and that a successful outcome can be achieved with clear-lens extraction. Our case also suggests that attention should be paid to the associations of drugs, including ritodrine, when APAC occurs with plateau iris.
